# New Phenolic Lipids from the Leaves of *Clausena harmandiana* Inhibit SARS-CoV-2 Entry into Host Cells

**DOI:** 10.3390/molecules28145414

**Published:** 2023-07-14

**Authors:** Marion Chambon, Charline Herrscher, Dana Al Halabi, Nathan François, Sandrine Belouzard, Stéphanie Boutet, Van Cuong Pham, Thi Mai Huong Doan, Karin Séron, Patrick Mavingui, Marc Litaudon, Chaker El Kalamouni, Cécile Apel

**Affiliations:** 1 Institut de Chimie des Substances Naturelles, CNRS, UPR 2301, Université Paris-Saclay, 91198 Gif-sur-Yvette, France; mllechambon@gmail.com; 2 Unité Mixte Processus Infectieux en Milieu Insulaire Tropical, Université de la Réunion, INSERM U1187, CNRS UMR 9192, IRD UMR 249, Plateforme Technologique CYROI, 94791 Sainte Clotilde, France; herrscher.charline@gmail.com (C.H.); danahalabi2899@gmail.com (D.A.H.); patrick.mavingui@cnrs.fr (P.M.); 3 Center for Infection and Immunity of Lille (CIIL), Institut Pasteur de Lille, Université de Lille, INSERM U1019, CNRS UMR 8204, CHU Lille, 59000 Lille, France; nathanfrancoislille@gmail.com (N.F.); sandrine.belouzard@ibl.cnrs.fr (S.B.); karin.seron@cnrs.fr (K.S.); 4 Institut Jean-Pierre Bourgin (IJPB), AgroParisTech, INRAE, Université Paris-Saclay, 78000 Versailles, France; stephanie.boutet@inrae.fr; 5 Institute of Marine Biochemistry, Vietnam Academy of Science and Technology (VAST), 18 Hoang Quoc Viet, CauGiay, Hanoi 10072, Vietnam; phamvc@yahoo.com (V.C.P.); doanthimaihuong0707@gmail.com (T.M.H.D.)

**Keywords:** *Clausena harmandiana*, phenolic lipids, antiviral activity, coronavirus

## Abstract

Induced by the spread of severe acute respiratory syndrome coronavirus 2 (SARS-CoV-2), the COVID-19 pandemic underlined the clear need for antivirals against coronaviruses. In an effort to identify new inhibitors of SARS-CoV-2, a screening of 824 extracts prepared from various parts of 400 plant species belonging to the Rutaceae and Annonaceae families was conducted using a cell-based HCoV-229E inhibition assay. Due to its significant activity, the ethyl acetate extract of the leaves of *Clausena harmandiana* was selected for further chemical and biological investigations. Mass spectrometry-guided fractionation afforded three undescribed phenolic lipids (**1**–**3**), whose structures were determined via spectroscopic analysis. The absolute configurations of **1** and **2** were determined by analyzing Mosher ester derivatives. The antiviral activity against SARS-CoV-2 was subsequently shown, with IC_50_ values of 0.20 and 0.05 µM for **2** and **3**, respectively. The mechanism of action was further assessed, showing that both **2** and **3** are inhibitors of coronavirus entry by acting directly on the viral particle. Phenolic lipids from *Clausena harmandiana* might be a source of new antiviral agents against human coronaviruses.

## 1. Introduction

In late 2019, a new coronavirus named SARS-CoV-2 (Severe Acute Respiratory Syndrome CoronaVirus 2) emerged in Wuhan, China, causing a global pandemic. Those with COVID-19 present symptoms of viral pneumonia, and the virus can cause fatal respiratory illness [1]. This novel coronavirus disease spread rapidly around the world, affecting tens of millions of people. Although vaccines have been developed, safe and efficient antiviral treatments are still needed to control the emergence of such viruses.

Recently, with the aim of discovering new antiviral agents, we have developed a molecular networking-based strategy that revolves around deciphering the relationship between spectral networks and biological activities and further exploiting such a relationship to prioritize the isolation of bioactive natural products [2,3,4]. In the present study, this approach allowed us to target the isolation of specific compounds from the ethyl acetate (EtOAc) extract of the leaves of *Clausena harmandiana* (associated with a strong inhibition of HCoV-229E replication).

*Clausena harmandiana* (Pierre) Pierre ex Guillaumin is a plant that belongs to the Rutaceae family and was harvested in north Vietnam. It is a small evergreen tree called “Song Fa” in Thai and is widely distributed in Southeast Asia. It is used in traditional medicines to relieve stomachache, headache, fever, eye-pain, and flatulence [5]. The major compounds found in its roots, root bark, stem bark, twigs, and fruit are carbazole alkaloids and coumarins [5]. Biological studies have shown that these types of molecules have antiviral properties against herpes simplex virus, hepatitis C virus, human immunodeficiency virus, human cytomegalovirus [6], influenza viruses, Enterovirus 71, coxsackievirus A16, dengue virus, and chikungunya virus [7]. They are also known for their cytotoxicity against several cancer cell lines [8] and their antibacterial, antifungal, and anti-malaria activity [9].

The present study describes the construction of a molecular network from LC-MS^2^ analyses of EtOAc *Clausena* spp. extracts, as well as mass spectrometry (MS)-guided isolation, structure elucidation, and the cytotoxic and antiviral (HCoV-229E and SARS-CoV-2) activities of three phenolic lipids (**1**–**3**) newly described from *C. harmandiana*. To the best of our knowledge, this is the first report on the phenolic lipids found in the genus *Clausena*.

## 2. Results and Discussion

Within a project aiming to investigate the potential antiviral activity of medicinal plants belonging to the Rutaceae and Annonaceae families, a total of 824 EtOAc plant extracts previously filtered on polyamide cartridge were screened for the inhibition of Human alphacoronavirus (HCoV-229E) as a coronavirus model. Huh-7 cells and Huh-7-TMPRSS2 cells were infected with a recombinant molecular clone expressing the luciferase gene reporter HCoV-229E-Luc, which allowed for rapid screening of the several hundred plant extracts. The collection of Rutaceae evaluated included eleven extracts from different plant parts of three species of the genus *Clausena*. Of these, only the crude EtOAc extract of the leaves of *C. harmandiana* showed potent antiviral activity; therefore, the crude EtOAc extract was selected for further chemical and biological investigations. 

**Figure 1:**
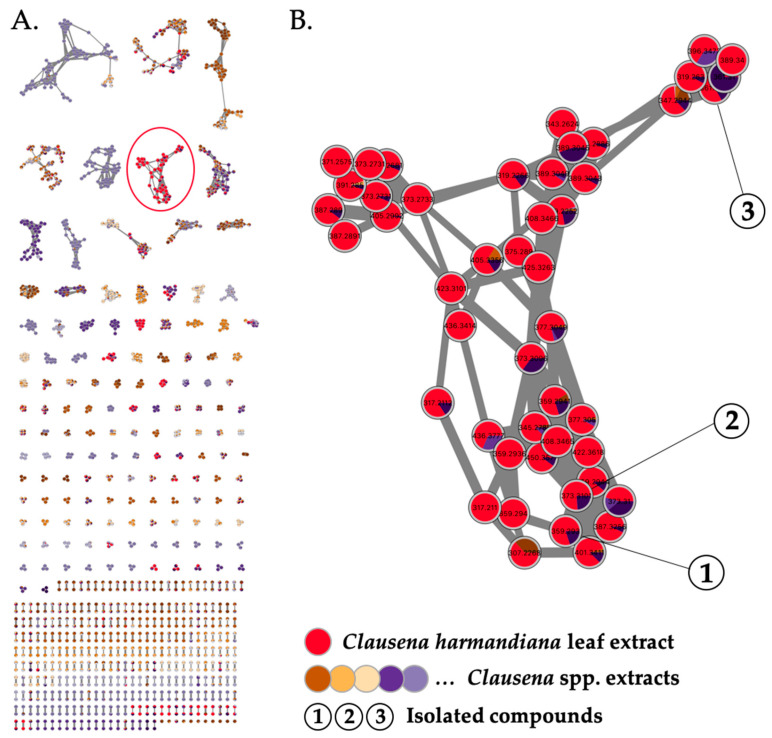
(**A**) Molecular network generated from LC-HRESIMS^2^ analysis of 11 EtOAc *Clausena* spp. extracts (self-loop nodes were removed from the network). (**B**) Detection of a cluster of ions specific to the active leaf extract of *Clausena harmandiana* (in red). Targeted isolation of compounds **1**–**3**.

**Figure 2:**
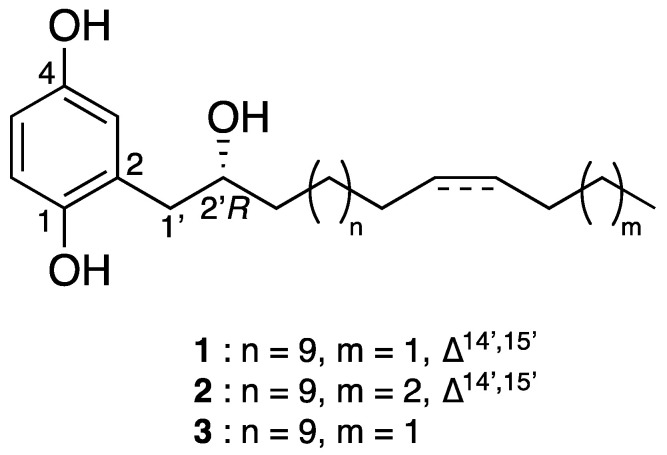
Chemical structures of phenolic lipids **1**–**3**.

All *Clausena* extracts were profiled by LC-HRESIMS^2^ as previously described [2,3,4]. Briefly, the samples were analyzed using a data-dependent acquisition mode. The resulting spectral data were preprocessed via MZmine 2 [10] and structured into molecular networks [11] using Metgem [12] (Figure 1A). The color code indicates which extract(s) each ion comes from. A cluster quasi-specific to the leaf extract of *C. harmandiana* (Figure 1B, in red) was highlighted. As the only active extract, the compounds corresponding to these ions were most likely responsible for the antiviral activity. Therefore, they were subjected to targeted isolation. The EtOAc crude extract was first subjected to silica gel flash column chromatography, yielding 11 fractions (F1–F11); subsequently, the fractions were analyzed using LC-HRESIMS. F4 were shown to contain compounds corresponding to the cluster of interest and, with an IC_50_ value of 0.06 µg/mL, displayed strong anti-HCoV-229E activity. From this fraction, C-18 column chromatography afforded the three undescribed phenolic lipids (referred to here as phenolic lipids **1–3**, the structures of which are shown in Figure 2).

### 2.1. Structural Elucidation

**Figure 3:**
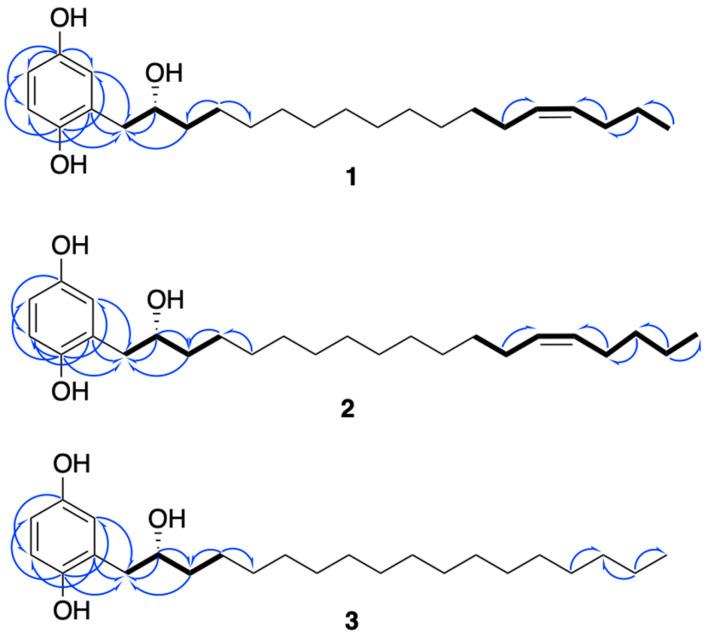
Key ^1^H-^1^H COSY (in bold) and HMBC (blue arrows) correlations of compounds **1**–**3**.

Compound **1** was obtained as a gray amorphous powder. Its molecular formula was defined as C_24_H_40_O_3_ by HRESIMS at *m*/*z* 359.2931 [M-H_2_O + H]^+^ (Calcd. for [C_24_H_39_O_2_]^+^, 359.2950), thus requiring five double bond equivalents. The ^1^H-NMR spectrum of **1** (Table 1) showed aromatic resonances for a 1,2,4-trisubstituted benzene ring [∂_H_ 6.77 (1H, d, *J* = 8.5 Hz, H-6), 6.60 (1H, dd, *J* = 2.7, 8.5 Hz, H-5), 6.54 (1H, d, *J* = 2.7 Hz, H-3)], resonances for a methyl group at ∂_H_ 0.90 (triplet, CH_3_-18′), a saturated carbon chain (∂_H_ 1.25–1.28), and two olefinic protons at ∂_H_ 5.36, suggesting the presence of an unsaturated alkyl chain. Analysis of the ^1^H-^1^H COSY spectrum revealed structural fragments from C-1′ to C-4′ and C-12′ to C-18′ (Figure 3). The signal at ∂_H_ 2.01 ppm corresponded to H-13′ and H-16′, the four protons adjacent to the double bond, and the signals observed as multiplets at ∂_H_ 2.75 and 3.96 were allocated to H_2_-1′ and H-2′, respectively. The ^13^C-NMR spectrum (Table 1) of compound **1** combined with HSQC spectrum showed carbon resonances for three non-protonated carbons—C-2, C-4, and C-1—at ∂_C_ 126.9, 149.4, and 149.6, respectively. This spectrum also showed six methines (∂_C_ 74.9, 115.1, 118.2, 118.4, 130.0, and 130.5), one methyl carbon at ∂_C_ 14.2, and several methylenes at 23.0–30.0 ppm. Analysis of these data suggested that the structure of **1** had an aromatic ring substituted by two hydroxy groups and a linear alkyl side chain bearing one hydroxy group and one double bond.

The positions of the three hydroxy groups at C-1, C-4, and C-2′ and the double bond at C-14′/C-15′ were confirmed by 2D NMR analyses (Figure 3). The para position of phenol groups was confirmed from the HMBC correlations from H-1′ (∂_H_ 2.75) to C-3 (∂_C_ 118.4); and H-3 (∂_H_ 6.77) to C-1′ (∂_C_ 39.2). In addition, the chemical shift values of the aromatic carbons are similar to those reported in the literature for close analogues [13]. The stereochemistry at the double bond between C-14′ and C-15′ was assigned as *Z* on the basis of allylic carbon resonances at ∂_C_ 27.6 and 29.6 ppm (C-13′ and C-16′) [14] and to similar values of coupling constants of structurally close alkenylresorcinols [15].

**Figure 4:**
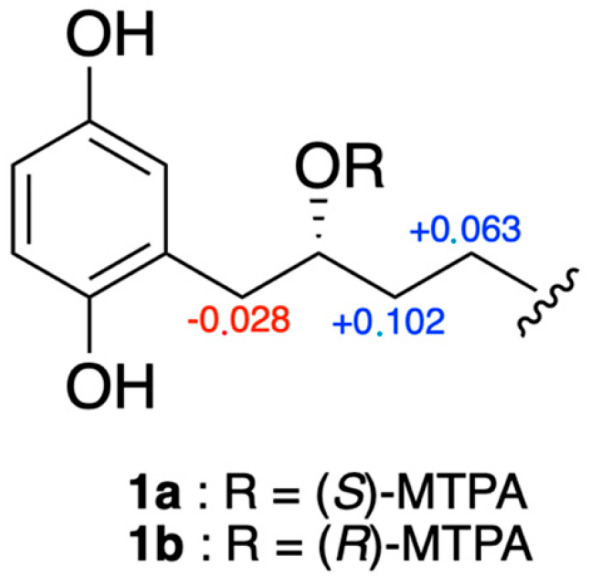
Δ*δ* values (ppm) for (*S*)- and (*R*)-MTPA esters of compounds **1a** and **1b**.

Due to the specific rotation value of 0 for compound **1**, we initially assumed that this compound could be a racemic mixture. However, it has been reported in the literature that choerosponols B and C, which possess similar planar structures and [α]_D_ values of 0, are enantiomerically pure [16]. Thus, to establish the absolute configuration of compound **1**, Mosher ester derivatization experiments were performed [17]. The secondary alcohol was converted into the (*S*)- and (*R*)-MPTA esters (**1a** and **1b**) following the procedure described in Brel et al. [18]. Based on the Δ*δ* (*δ_S_*_-_*δ_R_*) values of both MPTA esters (Figure 4), the *R* absolute configuration of C-2′ was determined.

Compound **2** possessed a molecular formula of C_25_H_42_O_3_ as deduced from HRESIMS at *m*/*z* 373.3112 [M-H_2_O + H]^+^ (Calcd. for [C_24_H_39_O_2_]^+^, 373.3107). NMR data were almost the same to those of **1** but suggested the presence of one additional methylene. Indeed, the ^13^C-NMR spectrum (Table 1) showed an extra carbon at 32.3 ppm. Its position was determined thanks to HMBC correlations (Figure 3) from H-17′ (∂_H_ 1.32) to C-18′ (∂_C_ 22.7) and C-16′ (∂_C_ 27.5). As for compound **1**, the configuration of the double bond was assigned as *Z*, with allylic carbon at ∂_C_ 27.3 and 27.5 (C-13′ and C-16′) and a coupling constant of 4.6 Hz. The analysis of the Mosher esters determined the absolute configuration of C-2′ as *R*. The chemical shift differences Δ*δ_S_*_-*R*_ of the neighboring protons were similar to those observed for compound **1** [H-1′ (−0.027 ppm), H-3′ (+0.131 ppm), H-4′ (+0.064 ppm)]. This compound was assigned as (2′*R*)-2-[(14′*Z*)-2′-hydroxynonadec-14′-en-1′-yl]benzene-1,4-diol and is the *R* enantiomer of choerosponol B [16].

Compound **3**, (2′*R*)-(2-(2-hydroxyoctadecyl)benzene-1,4-diol), possessed the molecular formula of C_24_H_42_O_3_, as deduced from conducting HRESIMS at *m*/*z* 361.3047 [M-H_2_O + H]^+^ (Calcd. for [C_24_H_39_O_2_]^+^, 361.3107). The NMR data of compound **3** were almost identical to those of **1** but without a double bond, as deduced from the absence of carbon around 130 ppm in the ^13^C-NMR spectrum (Table 1). Based on biogenetic considerations, the (*R*) absolute configuration of C-2′ was proposed, as for compounds **1** and **2**.

### 2.2. Cytotoxicity and H-CoV-229E Inhibition Assays

Compounds **1**–**3** were first evaluated for their cytotoxic activities against Huh7, a human hepatocyte cell line. All compounds showed cytotoxic activities in the micromolar range (CC_50_ between 0.5 and 1.3 µM) (Table 2). The maximum non-toxic concentration that provides more than 95% viability was 0.25 µM for all three tested compounds. This concentration was then used as the highest concentration for antiviral assays. Thus, to assess whether compounds **1**–**3** exert antiviral activity against HCoV-229E, Huh7 cells were infected with the recombinant molecular clone of HCoV-229E that expresses the luciferase (HCoV-229E-Luc) at the MOI of 0.5 in the presence of different concentrations (serial dilution starting from 0.25 µM) of each compound throughout the infection [19]. The results indicated that compound **1** showed no antiviral effect at non-cytotoxic doses, while compounds **2** and **3** exert dose-dependent antiviral activity at non-cytotoxic concentrations, with IC_50_ values of 0.1 and 0.05 µM, respectively, resulting in a selectivity index (SI) of 5 and 16 for **2** and **3**, respectively (Table 2). 

### 2.3. Cytotoxicity and SARS-CoV-2 Inhibition Assays

**Figure 5:**
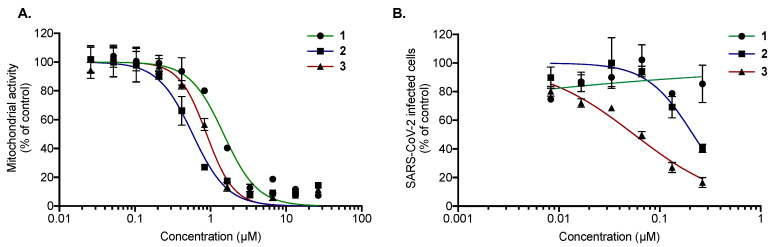
Compounds **2** and **3** exert antiviral activity against SARS-CoV-2 at non-cytotoxic concentrations. (**A**) Vero-E6 cells were treated with two-fold serial dilutions (25 to 0.02 µM) of **1**, **2**, or **3** for 48 h. Cell viability was evaluated through MTS assay. The results are means ± SD of three independent experiments and are expressed as relative value compared to the Mock-treated cells. (**B**) Vero-E6 cells were infected with SARS-CoV-2 at an MOI of 0.1 in the presence of different non-cytotoxic concentrations (0.25 to 0.01 µM) of each compound for 48 h, after which cells were fixed for 20 min with 3.7% PFA. Cells were then rinsed with PBS and processed for flow cytometric assay. Data are expressed relative to the Vehicle. The results are expressed as means ± SD of the three independent experiments.

Prior to the assessment of their antiviral activity against SARS-CoV-2, the cytotoxicity of compounds **1**–**3** was determined against Vero-E6 cells (Figure 5A). MTS assays showed that all tested compounds exert similar dose-dependent cytotoxicity on Vero-E6 cells with 50% cytotoxic concentration (CC_50_) values of 1.5 ± 0.20, 0.5 ± 0.08, and 0.9 ± 0.20 µM for **1**, **2**, and **3**, respectively (Figure 5A). Then, the antiviral activity of the three compounds was evaluated against SARS-CoV-2 in Vero-E6 cells. For this, Vero cells were infected with the pandemic strain of SARS-CoV-2 at an MOI of 0.1 for 48 h in the presence of different non-cytotoxic concentrations of the compounds (Figure 5B). The results showed that only **2** and **3** exert dose-dependent antiviral activity against SARS-CoV-2 at non cytotoxic concentrations (Figure 4B), with IC_50_ values of 0.20 ± 0.06 and 0.05 ± 0.03 µM, respectively. The selectivity index (SI) values—2.5 and 18 for **2** and **3**, respectively—are similar to those obtained with HCoV-229E. Taken together, these results showed that **3** is a strong inhibitor of SARS-CoV-2 infection. 

### 2.4. Characterization of the Antiviral Mechanism of Action of Compounds **2** and **3**

**Figure 6:**
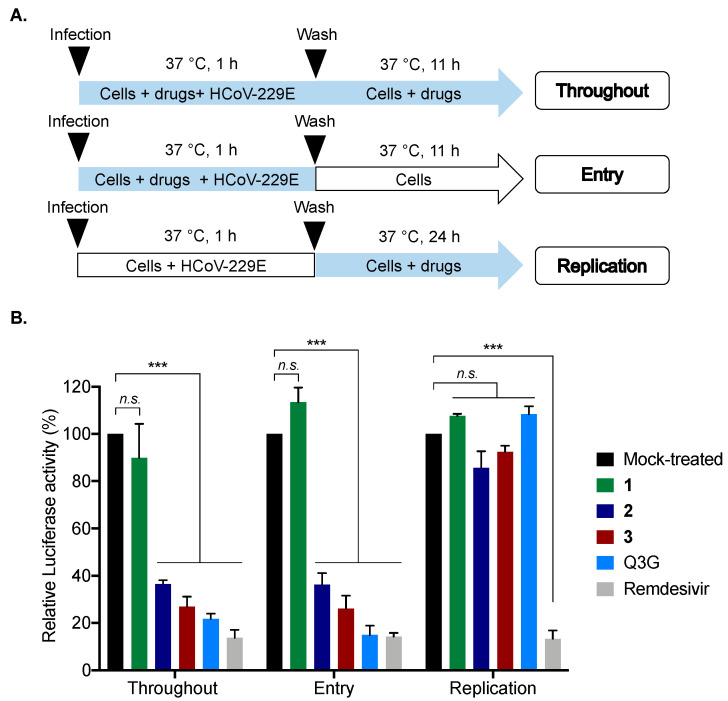
Compounds **2** and **3** inhibit HCoV-229E entry in human cells. (**A**) Schematic representation of time-of-drug addition approach performed to characterize the mechanism of action of compounds **2** and **3** (0.2 µM) in HCoV-229E-Luc-infected Huh7 cells. Q3G (25 µM) and Remdesivir (0.1 µM) were used as positive controls. Blue arrows indicate the presence of compounds. (**B**) Results of Luciferase activity in HCoV-229E-infected Huh7 cells under different conditions presented in panel **A**. The results are means ± SD of the three independent experiments and are expressed as relative value compared to the Mock-treated cells (Black column). One-way ANOVA and Dunnett’s test were used for statistical analysis (*** *p *< 0.001; *n.s.* = not significant).

To gain insights into the mechanism of action of **2** and **3** against coronaviruses, different experimental approaches were performed during HCoV-229E infection. Compounds were added at different stages of the viral replication cycle. To assess the impact on viral entry stage, HCoV-229E and compounds **2** and **3** were simultaneously co-added to the cells for 1 h (Figure 6A). Isoquercitrin (Q3G), which is known to inhibit the endocytic pathway, was used as a positive control [20], and compound **1** served as a negative control. To investigate whether **2** or **3** interfere with HCoV-229E replication, cells were first challenged with HCoV-229E for 1 h and then treated with **2** or **3** (Figure 6A). Remdesivir, which is known to inhibit virus replication, was used as a positive control [21].

Our data showed that no inhibition of infection was observed when **2** or **3** were added after virus inoculation, suggesting that **2** and **3** do not affect the virus replication step, unlike remdesivir (positive control), which strongly inhibited viral replication (Figure 6B). In contrast, strong inhibition of infection was noticed when **2** and **3** were present during the virus entry step (Figure 6B), as well as the positive control Q3G. Taken together, these results suggest that **2** and **3** act as virus entry inhibitors. 

**Figure 7:**
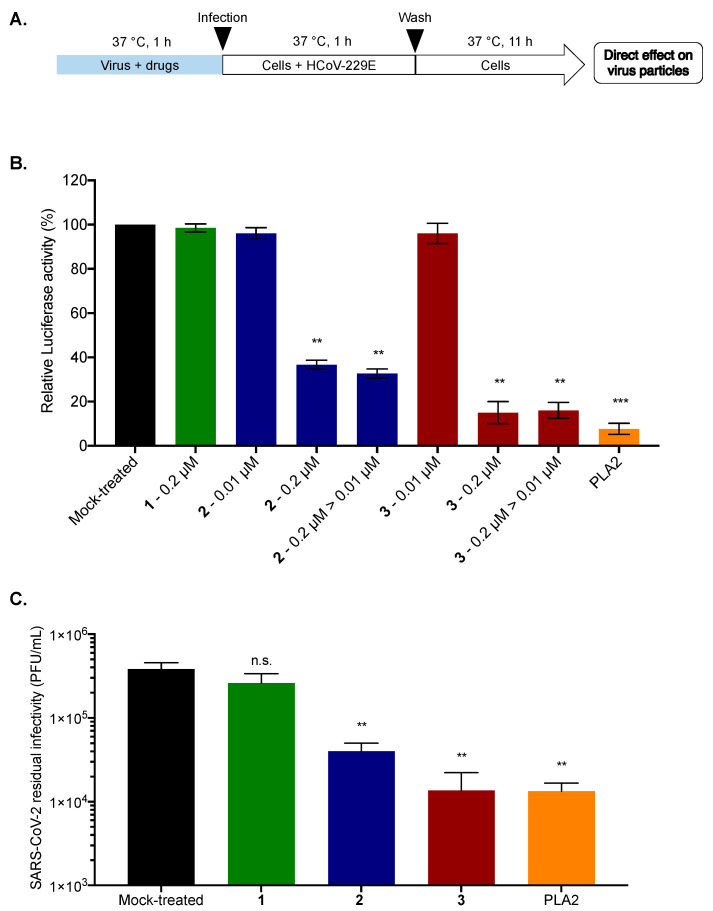
Compounds **2** and **3** inhibit viral entry by a direct action on the viral particle. (**A**) Schematic representation of free-virus particle assay to characterize the virucidal or virostatic effect of **2** and **3** (0.2 µM) against HCoV-229E-Luc. PLA2 (5 µg/mL) was used as a positive control. The amount of virus used for inoculation was identical for the different conditions; (**B**) Huh7 cells were infected with HCoV-229E-Luc in the presence of 0.2 or 0.01 µM of **2** or **3** or with HCoV-229E-Luc previously treated with 0.2 µM of **2** or **3** and then diluted 20 times, leading to a concentration of 0.01 µM of **2** or **3** for the incubation period (0.2 µM to 0.01 µM: 0.2 µM > 0.01 µM). At 12 h post-infection, the cells were lysed, and Luciferase activity was quantified. (**C**) SARS-CoV-2 inoculum was pre-incubated for 1 h with **1**, **2**, or **3** (0.2 µM) or 0.01% DMSO for the Mock-treated cells. PLA2 (5 µg/mL) was used as a positive control; The residual of infectivity was titrated on Vero-E6 cells. The results are means ± SD of three independent experiments and are expressed as relative value compared to the Mock-treated cells. One-way ANOVA and Dunnett’s test were used for statistical analysis (*** *p *< 0.001; ** *p *< 0.01; n.s. = not significant).

To further elucidate the underlying mechanism of antiviral action, we investigated whether **2** and **3** target the virus or the cells. HCoV-229E particles were pre-incubated with **2** or **3** (0.2 µM) for 1 h and then diluted 20-fold prior to infection to reach a concentration of 0.01 µM for inoculation (Figure 7A), a concentration that does not inhibit HCoV-229E-Luc infection for both **2** and **3** (as shown above). Compound **1** was used as a negative control. In parallel, Huh7 cells were infected with HCoV-229E-Luc and subsequently treated with 0.2 and 0.01 µM of **2** or **3**, serving as controls (Figure 7B). Phospholipase (PLA2), which is known to have broad-spectrum virucidal activity, was used as a positive control [22]. The results clearly indicated that when HCoV-229E-Luc was pre-incubated with **2** or **3** at a high concentration (0.2 µM) before infection at a low concentration (0.01 µM), the antiviral activity was much stronger than when infection was performed in the presence of 0.01 µM of **2** or **3** without pre-incubation (Figure 7B). Compounds **2** and **3** were able to inhibit the infection up to 60 and 80%, respectively, as well as PLA2, which inhibited 90% of infection (Figure 7B). Taken together, these results suggest that **2** and **3** inhibit HCoV229E entry by acting directly on the viral particle. 

To assess whether the antiviral activity of **2** and **3** against SARS-CoV-2 is also attributable to their ability to inhibit virus infectivity by acting directly on the virus particle, a residual infectivity assay was carried out. SARS-CoV-2 virus particles were incubated with **2** or **3** (0.2 µM). Compound **1** (0.2 µM) and PLA2 (5 µg/mL) were used as negative and positive controls, respectively. After 1 h of incubation, titration of residual infectivity showed that compounds **2** and **3** were able to decrease viral progeny up to 1 log as well as the positive control. Compound **3** appears to be the most effective at a non-cytotoxic dose of 0.2 µM, where the molecule is able to significantly inhibit SARS-CoV-2 infectivity by more than 1 log. These results suggest that compounds **2** and **3** possess antiviral activity against SARS-CoV-2 by acting directly on the virus particle. 

From a structural standpoint, compounds **1**–**3** are monosubstituted hydroquinone and belong to a large family of phenolic lipids. They are structurally very similar to choerosponols B and C isolated from *Choerospondias axillaris*, an Anacardiaceae native to Nepal [16]. They all possess a C-2′ hydroxylated unsaturated (**1** and **2**) or saturated (**3**) alkyl side chain, which only differs in the number of carbons: 18 for **1** and **3**; 19 for **2**. As choerosponols and with IC_50_ values in the micromolar range, compounds **1**–**3** exhibited strong cytotoxic activities on different cell lines. In contrast, only compounds **2** and **3** exert strong antiviral activities against HCoV-229E and SARS-CoV-2 viruses; compound **1** shows no antiviral activity. While the presence of a double bond in the C-14′ position of the alkyl side chain of compound **1** could explain the activity difference between compounds **1** and **3**, it cannot explain the difference regarding compound **2**, which only possesses an additional methylene group on the alkyl chain. Therefore, the discrepancy between the antiviral activity of **1** and **2** is difficult to explain rationally, and further studies are needed to explain it better.

Multi-informative molecular networks, which compare taxonomically related samples, are a highly useful approach for highlighting a chemical family of interest within a bioactive extract. In the present study, we used molecular networks in conjunction with biological data, which enabled us to directly target the isolation of two compounds with strong antiviral activities.

## 3. Materials and Methods

### 3.1. Plant Material 

The leaves of *Clausena harmandiana* were collected in Hòa Bình, Mai Châu, Vietnam, in April 1996 and identified by Dr. Nguyen Cuong. A voucher specimen (VN-0081) was deposited at the Institute of Ecology and Biological Resources, Vietnam Academy of Science and Technology (VAST), Hanoï.

### 3.2. Phytochemical Analysis—General Experimental Procedures

Optical rotations were measured at 24 °C on an MCP 300 polarimeter (Anton Paar, Les Ulis, France). UV spectra were recorded using a Varian Cary 100 UV–vis spectrophotometer. IR spectra were recorded using a PerkinElmer BX FT-IR spectrometer. All NMR spectra were recorded using a 300 MHz instrument (Avance 300, Bruker, Wissembourg, France). Chemical shifts (relative to CDCl_3_ or acetone for 3) are in ppm, and coupling constants (*J*) are in Hz. The multiplicity of signals is reported as follows: s, singlet; d, doublet; dd, doublet of doublets; t, triplet; m, multiplet. HR-ESI-MS were run on an ESI-TOF spectrometer (LCT, Waters, Guyancourt, France). Nucleodur analytical and preparative C-18 columns (250 mm × 4.6 mm and 250 mm × 21 mm; 5 µm Macherey-Nagel, Hoerdt, France), were used for preparative HPLC separations using a Waters autopurification system equipped with a sample manager (Waters 2767), a column fluidics organizer, a binary pump (Waters 2525), a UV–Vis diode array detector (190–600 nm, Waters 2996), and a PL-ELS 1000 ELSD Polymer Laboratory detector. A prepacked puriFlash (Intershim, Montluçon, France) silica cartridge was used for flash chromatography using a Combiflash Rf 200i (Teledyne Isco, Lincoln, NE, USA). For thin-layer chromatography (TLC), pre-coated silica gel 60 F254 (0.25 mm, Merck, Saint Quentin Fallavier, France) plates were used. All other chemicals and solvents were purchased from Carlo Erba (Val de Reuil, France).

### 3.3. Extraction and Isolation

Dried leaves of *C. harmandiana* (150 g) were extracted with EtOAc (3 × 200 mL, 1 h each at room temperature). The EtOAc solutions were combined and evaporated to dryness under reduced pressure to give a crude residue (1.2 g). This residue was subjected to flash chromatography over silica gel and eluted with a gradient of Heptane-EtOAc (90:10 to 0:100) then EtOAc-MeOH (95:5 to 80:20) to yield 11 fractions (F1–F11). F4 (151 mg) was further purified by preparative HPLC (C-18 column, Nucleodur, 250 mm × 21 mm, 5 µm, CH_3_CN-H_2_O 90:10 + 0.1% formic acid at 21 mL/min) to yield compounds **1** (34.0 mg; t_R_ 13.6 min), **2** (63.3 mg; t_R_ 17.4 min), and **3** (10.6 mg; t_R_ 23.4 min).

#### 3.3.1. (2′. R)-2-[(14′Z)-2′-Hydroxyoctadec-14′-en-1′-yl]benzene-1,4-diol (**1**)

Gray amorphous powder. ${\left[\alpha \right]}_{D}^{24}$ ± 0.0 (*c* 1, CHCl_3_); UV (MeOH) *λ*_max_ (log *ε*), 294 nm (3.7), 202 nm (4.5); IR *ν*_max_ 3190, 2919, 1458, 1195, 1012, 812, and 722 cm^−1^. HRESIMS *m*/*z*: 359.2931 [M-H_2_O + H]^+^ (Calcd. for C_24_H_39_O_2_^+^, 359.2950). ^1^H-NMR (CDCl_3_, 300 MHz) and ^13^C-NMR (CDCl_3_, 300 MHz); see Table 1.

#### 3.3.2. (2′. R)-2-[(14′Z)-2′-Hydroxynonadec-14′-en-1′-yl]benzene-1,4-diol (**2**)

Beige amorphous powder. ${\left[\alpha \right]}_{D}^{24}$ ± 0.0 (*c* 1, CHCl_3_); UV (MeOH) *λ*_max_ (log *ε*), 293 nm (3.1), 201 nm (3.9); IR *ν*_max_ 3355, 2920, 1451, 1197, 1101, 814, and 721 cm^−1^. HRESIMS *m*/*z*: 373.3112 [M-H_2_O + H]^+^ (Calcd. for C_25_H_41_O_2_^+^, 373.3107). ^1^H-NMR (CDCl_3_, 300 MHz) and ^13^C-NMR (CDCl_3_, 300 MHz); see Table 1.

#### 3.3.3. (2′. R)-(2-(2-Hydroxyoctadecyl)benzene-1,4-diol) (**3**)

Beige amorphous powder. ${\left[\alpha \right]}_{D}^{24}$ + 2.0 (*c* 1, acetone); UV (MeOH) *λ*_max_ (log *ε*), 294 nm (3.5), 202 nm (4.3); IR *ν*_max_ 3181, 2918, 1463, 1206, 1101, 822, and 719 cm^−1^. HRESIMS *m*/*z*: 361.3047 [M-H_2_O + H]^+^ (Calcd. for C_24_H_41_O_2_^+^, 361.3107). ^1^H-NMR (Acetone-*d*6, 300 MHz) and ^13^C-NMR (Acetone-*d*6, 300 MHz); see Table 1.

### 3.4. Preparation of (S)-MTPA and (R)-MTPA Esters of **1** and **2**

In an NMR sample tube, 1.0 mg of dimethylaminopyridine (DMAP) was added to 1.0 mg of compound **1** or **2**. Additionally, 9 μL of pyridine-*d*_5_ and 600 μL of CDCl_3_ were added to the mixtures. Then, (*R*)-MTPA chloride (5 µL, 27 μmol) was added. The resultant reaction mixtures were stirred at RT for 2 h to produce (*S*)-MTPA esters **1a** and **2a**. The identical procedure was carried out to obtain the (*R*)-MTPA esters **1b** and **2b** from (*S*)-MTPA chloride.

(*S*)-MTPA Ester (**1a**). ^1^H NMR (500 MHz, CDCl_3_): 0.91 (3H, t, H-18′), 1.34 (2H, m, H-17′), 2.02 (4H, m, H-13′, H-16′), 1.27 (16H, m, H-5′-12′), 5.36 (2H, m, H-14′, H-15′), 2.67 (2H, m, H-1′), 5.21 (1H, m, H-2′), 1.46 (2H, m, H-3′), 1.21 (2H, m, H-4′), 3.25, 3.43 (3H, s, MTPA-OCH_3_).

(*R*)-MTPA Ester (**1b**). ^1^H NMR (500 MHz, CDCl_3_): 0.91 (3H, t, H-18′), 1.34 (2H, m, H-17′), 2.02 (4H, m, H-13′, H-16′), 1.27 (16H, m, H-5′-12′), 5.37 (2H, m, H-14′, H-15′), 2.70 (2H, m, H-1′), 5.22 (1H, m, H-2′) 1.35 (2H, m, H-3′), 1.15 (2H, m, H-4′), 3.26, 3.43 (3H, s, MTPA-OCH_3_).

(*S*)-MTPA Ester (**2a**). ^1^H NMR (500 MHz, CDCl_3_): 0.82 (3H, t, H-19′), 1.25 (4H, m, H-17′, H-18′), 1.95 (4H, m, H-13′, H-16′), 1.19 (16H, m, H-5′-12′), 5.28 (2H, m, H-14′, H-15′), 2.58 (2H, m, H-1′), 5.13 (1H, m, H-2′), 1.37 (2H, m, H-3′), 1.13 (2H, m, H-4′), 3.17, 3.34 (3H, s, MTPA-OCH_3_).

(*R*)-MTPA Ester (**2b**). ^1^H NMR (500 MHz, CDCl_3_): 0.82 (3H, t, H-19′), 1.25 (4H, m, H-17′, H-18′), 1.95 (4H, m, H-13′, H-16′), 1.19 (16H, m, H-5′-12′), 5.28 (2H, m, H-14′, H-15′), 2.61 (2H, m, H-1′), 5.14 (1H, m, H-2′), 1.24 (2H, m, H-3′), 1.07 (2H, m, H-4′), 3.17, 3.34 (3H, s, MTPA-OCH_3_).

### 3.5. Data-Dependent LC-ESI-HRMS^2^ Analysis

LC analyses were performed using a Thermo Ultimate 3000 system equipped with a Cortecs C_18_ column (2.1 × 100 mm; 2.7 μm, Waters). The mobile phase consisted of water-acetonitrile (H_2_O-CH_3_CN) acidified with 0.1% formic acid (90:10) held for 2 min, then a gradient from 90:10 to 0:100 in 20 min held at 0:100 for 8 min at a flow rate of 600 µL.min^−1^. The temperature of the column oven was set to 40 °C, and the injection volume was set to 5 µL. LC-ESI-HRMS^2^ analyses were achieved by coupling the LC system to an Impact II Bruker quadrupole time-of-flight mass spectrometer (Bruker Daltonics, Bremen, Germany) equipped with an ESI dual source (operating in the positive-ion mode). Source parameters were set as follows: end plate offset—350 V, capillary voltage—4500 V, nebulizer pressure—60 psi, drying gas flow rate—10 L.min^−1^, drying gas temperature—240 °C. MS scans were operated in full-scan mode from *m*/*z* 100 to 1400 (at 6 Hz). MS^1^ scan was followed by MS^2^ scans of the five most intense ions above an absolute threshold of 2000 counts. Selected parent ions were fragmented with a collision energy fixed at 30 eV and using an *m*/*z* dependent isolation window of 2–4 amu**.** The mass accuracy was guaranteed via an injection of a calibration solution from sodium formate clusters with external (at the beginning of each run) and internal (segment 0.1 at 0.4 min of each sample) calibration by a High Precision Calibration (HPC) equation with a maximum mass delta of 1 ppm and 7 as the minimal number of calibration points. LC-UV and MS data acquisition and processing were performed using DataAnalysis 4.4 software (Bruker Daltonics, Bremen, Germany).

### 3.6. MZmine 2 Pre-Processing

The MS^2^ data files were converted from the .d Agilent standard data format to the .mzXML format using MSConvert software (part of the ProteoWizard package (Palo Alto, CA, USA, v3)) [23]. The .d Bruker data files were converted to .mzXML format using DataAnalysis 4.4 software. All .mzXML were then processed using MZmine 2 v53 [10]. Mass detection was conducted using a noise level of 400 counts for MS and 0 count for MSMS dimension. The ADAP chromatogram builder was used with a minimum group size of scans of 4, a group intensity threshold of 3000, a minimum highest intensity of 4000, and an *m*/*z* tolerance of 15 ppm [24]. The ADAP wavelets deconvolution algorithm was used with the following standard settings: S/N threshold = 8, minimum feature height = 3000, coefficient/area threshold = 10, peak duration range—0.02–1.0 min, RT wavelet range—0.01–0.07. Isotopologues were grouped using the isotopic peaks grouper algorithm, with an *m*/*z* tolerance of 15 ppm and an RT tolerance of 0.1 min. MS^2^ scans were paired using an *m*/*z* tolerance range of 0.025 Da and RT tolerance range of 0.1 min. Peak alignment was performed using the join aligner module (*m*/*z* tolerance = 15 ppm, weight for *m*/*z* = 1, weight for RT = 1, absolute RT tolerance = 0.1 min). The peak list was gap-filled with the peak finder module (*m*/*z* tolerance = 5 ppm and RT tolerance = 0.05 min). Eventually, the .mgf spectral data file and its corresponding .csv metadata file (containing RT and peak areas) were exported using the dedicated “Export to GNPS-FBMN” built-in module [11,25].

### 3.7. Molecular Network Analysis

The two files mentioned above were imported into MetGem 1.3.6. [12]. MS^2^ spectra were window-filtered by choosing only the top ten peaks within the ±50 Da window throughout the spectrum. The data were filtered by removing all peaks in the ±5 Da range around the precursor *m*/*z*. The *m*/*z* tolerance window used to find the matching peaks was set to 0.02 Da, and cosine scores were kept under consideration for spectra sharing at least 4 matching peaks. The network was created where edges were filtered to have a cosine score above 0.8. Further edges between two nodes were kept in the network only if each of the nodes appeared in each other’s respective top 10 most similar nodes.

### 3.8. Cells and Culture Conditions

Human-derived hepatoma cells, Huh7 (ATCC, PTA-8561) cells, and Vero-E6 cells were grown in Dulbecco’s Modified Eagle Medium (DMEM, PAN Biotech, Aidenbach, Germany) supplemented with 10% heat-inactivated fetal bovine serum (FBS), 1% penicillin/streptomycin, and 0.1% amphotericin B (PAN Biotech). Cells were cultured at 37 °C in 5% CO_2_ incubator. 

### 3.9. Viruses

Recombinant HCoV-229E-Luc expressing the luciferase gene was kindly provided by V. Thiel [26]. SARS-CoV-2 virus was isolated in 2020 from a nasopharyngeal swab of a COVID-19 PCR-positive patient in Reunion Island [27]. SARS-CoV-2 was propagated on Vero-E6 cells. 

### 3.10. Chemicals and Antibody

Q3G, PLA2 were purchased from Sigma-Aldrich (Saint-Quentin-Fallavier, France). Remdesivir was purchased from Invivogen (Toulouse, France). All stock solutions were prepared in sterile dimethyl sulfoxide, DMSO (Sigma-Aldrich). Monoclonal human igG1 antibody SARS-CoV-2 spike (clone H4) was purchased from Invivogen (Toulouse, France).

### 3.11. Cytotoxic Assays

The cytotoxicity of isolated phytocompounds were determined using an MTS [3-(4,5-dimethylthiazol-2-yl)-5-(3-carboxymethoxyphenyl)-2-(4-sulfophenyl)-2H-tetrazolium]-based viability assay (CellTiter Aqueous One Solution Cell Proliferation Assay from Promega, Charbonnières-les-Bains, France). A total of 2 × 10^4^ Huh7 and Vero-E6 cells were seeded on a 96-well plate and incubated with a serial dilution of phytocompounds. Forty-eight hours after treatment, the MTS test was performed according to the manufacturer’s instructions. Absorbance was assessed at 490 nm, and the percentage of viable cells was calculated. Dose–response curves were established on Prism GraphPad to calculate the concentration causing death in 50% of the cells (CC_50_). 

### 3.12. HCoV-229E-Luc Infection Inhibition Assays (Screening of 824 Plant Extracts)

HCoV-229E-Luc was mixed with the plant extracts at three different concentrations (25, 10 and 2.5 µg/mL) for 10 min. Huh-7 cells and Huh-7 cells transduced with a lentiviral vector expressing the TMPRSS2 protease gene (Huh-7-TMPRSS2 cells) were infected with HCoV-229E-Luc at a multiplicity of infection (MOI) of 0.5 in a final volume of 50 μL for 1 h at 37 °C in the presence of the plant extracts. The virus was removed and replaced with culture medium containing the extracts for 6 h at 37 °C. Cells were lysed in 20 μL of Renilla lysis buffer (Promega), and luciferase activity was quantified in a Tristar LB 941 luminometer (Berthold Technologies, Bad Wildbad, Germany) using a Renilla luciferase assay system (Promega), as recommended by the manufacturer.

### 3.13. HCoV-229E-Luc Infection Inhibition Assays (Evaluation of Fractions and Pure Compounds)

Huh7 cells were treated with different concentrations of fractions or compounds diluted in the culture medium and inoculated with HCoV-229E at a MOI of 0.5 in a final volume of 100 μL. At 12 h post-infection, the medium was removed, and the cells were lysed in 40 μL of Renilla luciferase buffer (Promega, E2810). Luminescence was measured using the FLUOstar Omega spectrophotometer and by following the manufacturer’s instructions. 

### 3.14. SARS-CoV-2 Infection Inhibition Assays 

Vero-E6 cells were seeded in 24-well plates overnight before inoculation with SARS-CoV-2 at an MOI of 0.1 in the presence of **1**–**3** at different concentrations for 48 h at 37 °C. Vero-E6 cells were trypsinized and fixed for 20 min with 3.7% PFA. The cells were then rinsed with PBS and processed for flow cytometric assay, Cytoflex (Beckman, Villepinte, France), as previously described using monoclonal human igG1 antibody SARS-CoV-2 spike (clone H4) for 1 h, followed by a Goat anti-Human IgG Cross-Adsorbed secondary antibody, Alexa Fluor 488 (Thermo Fisher Scientific, Waltham, MA, USA), for the detection of infected cells. The percentage of infected cells was assessed using Cytexpert software (version 9.00, La Jolla, CA, USA).

### 3.15. Virucidal Assay against SARS-CoV-2

Moreover, 25 × 10^4^ PFU of SARS-CoV-2 were pre-incubated with the drugs in DMEM at 37 °C for 1 h; DMSO (0.01%) was used as vehicle control. After pre-incubation, the mixture was diluted (serial tenfold dilution) and used to infect a monolayer of Vero-E6 cells. After incubation at 37 °C for 1 h, the infected cells were covered with 300 µL of medium containing 1.0% carboxymethylcellulose (CMC) with DMEM + 5% FBS. The cells were incubated at 37 °C for 3 days. Finally, the Vero-E6 cells were fixed with 3.7% PFA and stained with Crystal violet. Plaques were counted and compared to the control. 

### 3.16. Statistical Analysis and IC_50_ and CC_50_ Determination 

Both the Cytotoxic concentrations (CC_50_) and inhibitory concentrations (IC_50_) were obtained by performing nonlinear regression followed by the construction of sigmoidal concentration–response curves. The results are means ± SD of at least three independent experiments performed in triplicate and are expressed as relative value compared to untreated infected cells (Mock-treated). Statistical analyses were conducted using one-way ANOVA. All statistical analyses were carried out using GraphPad Prism software (version 9.0; La Jolla, CA, USA). Significance levels are shown in the figures as follows: * *p* < 0.05; ** *p* < 0.01; *** *p* < 0.001, **** *p* < 0.0001, n.s. = not significant.

## Figures and Tables

**Table 1:** ^1^H NMR (300 MHz) and ^13^C NMR (75 MHz) of compounds **1**–**3** in CDCl_3_ (**1** and **2**) and acetone-*d*6 (**3**).

	1		2		3	
No.	* δ *_C_	*δ*H	* δ *_C_	*δ*H	* δ *_C_	*δ*H
1-OH	149.6	-	149.6	-	149.6	-
2	126.9	-	127.0	-	127.5	-
3	118.4	6.54 (d, *J* = 2.7)	118.4	6.54 (d, *J* = 2.9)	118.6	6.63 (d, *J* = 2.7)
4-OH	149.4	-	149.3	-	150.8	-
5	115.1	6.60 (dd, *J* = 2.7, 8.5)	115.1	6.61 (dd, *J* = 2.9, 8.5)	114.5	6.58 (dd, *J* = 2.7, 8.5)
6	118.2	6.77 (d, *J* = 8.5)	118.2	6.78 (d, *J* = 8.5)	117.3	6.67 (d, *J* = 8.5)
1′	39.2	2.75 (m)	39.2	2.75 (m)	39.8	2.75 (m)
2′	74.9	3.96 (m)	74.9	3.96 (m)	73.3	3.95 (m)
3′	37.3	1.50 (m)	37.3	1.50 (m)	37.6	1.52 (m)
4′	26.0	1.37 (m)	26.0	1.35 (m)	26.2	1.40 (m)
5′–12′	29.6–30.1	1.25–1.28	29.7–30.1	1.27–1.29	29.1–30.1	1.33
13′	27.6	2.01 (m)	27.3	2.02 (m)	30.1	1.33
14′	130.5	5.36 (t, *J* = 5.4)	130.2	5.35 (t, *J* = 4.6)	30.2	1.33
15′	130.0	5.36 (t, *J* = 5.4)	130.2	5.35 (t, *J* = 4.6)	30.2	1.33
16′	29.6	2.01 (m)	27.5	2.02 (m)	32.4	1.33
17′	23.2	1.37 (m)	32.3	1.32 (m)	23.1	1.33
18′	14.2	0.90 (t)	22.7	1.32 (m)	14.1	0.92 (t)
19′			14.3	0.90 (t)		

**Table 2:** Cytotoxicity and antiviral activity against HCoV-229E of **1**, **2**, and **3**.

Compound	CC_50_ (µM) ^a^	IC_50_ (µM) ^b^	SI ^c^
**1**	1.30 ± 0.10	na	-
**2**	0.50 ± 0.05	0.10 ± 0.03	5
**3**	0.80 ± 0.10	0.05 ± 0.04	16

Cytotoxic concentration (CC_50_) and inhibitory concentration (IC_50_) were obtained by performing nonlinear regression followed by the construction of the sigmoidal concentration–response curves from Figure 4. ^a^ Concentration inhibited cell viability by 50%; ^b^ Concentration inhibited infection by 50%; ^c^ Selectivity index (CC_50_/IC_50_). na: non active.

## Data Availability

The data presented in this study are available on request from the corresponding authors. The LC-HRESIMS^2^ data are not yet publicly available due to their use in other ongoing research projects.

## Sample Availability

Samples of the compounds are available from the authors.

## References

[1] Hu B., Guo H., Zhou P., Shi Z.L. (2021). Characteristics of SARS-CoV-2 and COVID-19. Nat. Rev. Microbiol..

[2] Olivon F., Allard P.M., Koval A., Righi D., Genta-Jouve G., Neyts J., Apel C., Pannecouque C., Nothias L.F., Cachet X. (2017). Bioactive Natural Products Prioritization Using Massive Multi-Informational Molecular Networks. ACS Chem. Biol..

[3] Olivon F., Apel C., Retailleau P., Allard P.M., Wolfender J.L., Touboul D., Roussi F., Litaudon M., Desrat S. (2018). Searching for Original Products by Molecular Networking: Detection, Isolation and Total Synthesis of Chloroaustralasines. Org. Chem. Front..

[4] Olivon F., Remy S., Grelier G., Apel C., Eydoux C., Guillemot J.C., Neyts J., Delang L., Touboul D., Roussi F. (2019). Antiviral Compounds from *Codiaeum peltatum* Targeted by a Multi-informative Molecular Networks Approach. J. Nat. Prod..

[5] Jantamat P., Weerapreeyakul N., Puthongking P. (2019). Cytotoxicity and Apoptosis Induction of Coumarins and Carbazole Alkaloids from *Clausena harmandiana*. Molecules.

[6] Caruso A., Ceramella J., Iacopetta D., Saturnino C., Mauro M.V., Bruno R., Aquaro S., Sinicropi M.S. (2019). Carbazole Derivatives as Antiviral Agents: An Overview. Molecules.

[7] Mishra S., Pandey A., Manvati S. (2020). Coumarin: An emerging antiviral agent. Heliyon.

[8] Songsiang U., Thongthoom T., Boonyarat C., Yenjai C. (2011). Claurailas A–D, cytotoxic carbazole alkaloids from the roots of *Clausena harmandiana*. J. Nat. Prod..

[9] Arbab I.A., Abdul A.B., Aspollah M., Abdelwahab S.I., Ibrahim M.Y., Ali Z. (2012). A review of traditional uses, phytochemical and pharmacological aspects of selected members of *Clausena* genus (Rutaceae). J. Med. Plant. Res..

[10] Pluskal T., Castillo S., Villar-Briones A., Oresic M. (2010). MZmine 2: Modular framework for processing, visualizing, and analyzing mass spectrometry-based molecular profile data. BMC Bioinform..

[11] Wang M., Carver J.J., Phelan V.V., Sanchez L.M., Garg N., Peng Y., Nguyen D.D., Watrous J., Kapono C.A., Luzzatto-Knaan T. (2016). Sharing and community curation of mass spectrometry data with Global Natural Products Social Molecular Networking. Nat. Biotechnol..

[12] Olivon F., Elie N., Grelier G., Roussi F., Litaudon M., Touboul D. (2018). MetGem Software for the Generation of Molecular Networks Based on the t-SNE Algorithm. Anal. Chem..

[13] Groweiss A., Cardellina J.H., Pannell L.K., Uyakul D., Kashman Y., Boyd M.R. (1997). Novel cytotoxic, alkylated hydroquinones from *Lannea welwitschii*. J. Nat. Prod..

[14] Rossi R., Carpita A., Quirici M.G., Veracini C.A. (1982). Insect pheromone components: Use of 13C NMR spectroscopy for assigning the configuration of C=C double bonds of monoenic or dienic pheromone components and for quantitative determination of *Z*/*E* mixtures. Tetrahedron.

[15] Fürstner A., Seidel G. (1997). Shortcut Syntheses of Naturally Occurring 5-Alkylresorcinols with DNA-Cleaving Properties. J. Org. Chem..

[16] Kil Y.S., Risinger A.L., Petersen C.L., Liang H., Grkovic T., O’Keefe B.R., Mooberry S.L., Cichewicz R.H. (2020). Using the Cancer Dependency Map to Identify the Mechanism of Action of a Cytotoxic Alkenyl Derivative from the Fruit of *Choerospondias axillaris*. J. Nat. Prod..

[17] Ohtani I., Kusumi T., Kashman Y., Kakisawa H. (1991). High-field FT NMR application of Mosher’s method. The absolute configurations of marine terpenoids. J. Am. Chem. Soc..

[18] Brel O., Touré S., Levasseur M., Lechat C., Pellissier L., Wolfender J.L., Van-Elslande E., Litaudon M., Dusfour I., Stien D. (2020). Paecilosetin Derivatives as Potent Antimicrobial Agents from *Isaria farinosa*. J. Nat. Prod..

[19] Meunier T., Desmarets L., Bordage S., Bamba M., Hervouet K., Rouillé Y., François N., Decossas M., Sencio V., Trottein F. (2022). A Photoactivable Natural Product with Broad Antiviral Activity against Enveloped Viruses, Including Highly Pathogenic Coronaviruses. Antimicrob. Agents Chemother..

[20] Gaudry A., Bos S., Viranaicken W., Roche M., Krejbich-Trotot P., Gadea G., Desprès P., El-Kalamouni C. (2018). The Flavonoid Isoquercitrin Precludes Initiation of Zika Virus Infection in Human Cells. Int. J. Mol. Sci..

[21] Parang K., El-Sayed N.S., Kazeminy A.J., Tiwari R.K. (2020). Comparative Antiviral Activity of Remdesivir and Anti-HIV Nucleoside Analogs against Human Coronavirus 229E (HCoV-229E). Molecules.

[22] Chen M., Aoki-Utsubo C., Kameoka M., Deng L., Terada Y., Kamitani W., Sato K., Koyanagi Y., Hijikata M., Shindo K. (2017). Broad-spectrum antiviral agents: Secreted phospholipase A(2) targets viral envelope lipid bilayers derived from the endoplasmic reticulum membrane. Sci. Rep..

[23] Chambers M.C., Maclean B., Burke R., Amodei D., Ruderman D.L., Neumann S., Gatto L., Fischer B., Pratt B., Egertson J. (2012). A cross-platform toolkit for mass spectrometry and proteomics. Nat. Biotechnol..

[24] Myers O.D., Sumner S.J., Li S., Barnes S., Du X. (2017). One Step Forward for Reducing False Positive and False Negative Compound Identifications from Mass Spectrometry Metabolomics Data: New Algorithms for Constructing Extracted Ion Chromatograms and Detecting Chromatographic Peaks. Anal. Chem..

[25] Nothias L.F., Petras D., Schmid R., Dührkop K., Rainer J., Sarvepalli A., Protsyuk I., Ernst M., Tsugawa H., Fleischauer M. (2020). Feature-based molecular networking in the GNPS analysis environment. Nat. Methods.

[26] Van den Worm S.H., Eriksson K.K., Zevenhoven J.C., Weber F., Züst R., Kuri T., Dijkman R., Chang G., Siddell S.G., Snijder E.J. (2012). Reverse genetics of SARS-related coronavirus using vaccinia virus-based recombination. PLoS ONE.

[27] Wilkinson D.A., Mercier A., Turpin M., Simbi M.A., Turpin J., Lebarbenchon C., Cesari M., Jaffar-Bandjee M.C., Josset L., Yemadje-Menudier L. (2022). Genomic evolution of SARS-CoV-2 in Reunion Island. Infect. Genet. Evol..

